# Identification of antibacterial substances of *Lactobacillus plantarum* DY‐6 for bacteriostatic action

**DOI:** 10.1002/fsn3.1585

**Published:** 2020-05-12

**Authors:** Yin Mao, Xiaojuan Zhang, Zhenghong Xu

**Affiliations:** ^1^ National Engineering Laboratory for Cereal Fermentation Technology (NELCF) School of Biotechnology Jiangnan University Wuxi China; ^2^ Jiangsu Provincial Research Center for Bioactive Product Processing Technology Jiangnan University Wuxi China

**Keywords:** antibacterial substances, bacteriostatic mechanism, *Lactobacillus plantarum* DY‐6, metabolomics, statistical analysis

## Abstract

The antimicrobial activity of lactic acid bacteria is closely related to its metabolites. Our results showed that *Lactobacillus plantarum* DY‐6 had the highest antibacterial activities among the seven bacteria tested in this study. To fully understand the active antimicrobial substances in *L. plantarum* DY‐6, the cell‐free supernatant (CFS) were analyzed. Our data indicated that the antibacterial effect of the CFS was positively correlated with the growth of the bacteria, and the main antibacterial substances were lactic acid, acetic acid, propionic acid, caprylic acid, and decyl acid. Finally, this study demonstrated that the antibacterial active substance produced by the lactic acid bacteria could destroy the cell membrane structure of the bacteria, causing bacteria to fail to grow and reproduce normally, thereby exerting a bacteriostatic action. Taken together, our current findings would provide an effective method for rapid screening of antimicrobial substances.

## Graphical abstract

The main antibacterial substances were found to be lactic acid, acetic acid, propionic acid, caprylic acid and decyl acid. The antibacterial active substance produced by the lactic acid bacteria was found to destroy the cell membrane structure of the bacteria, causing bacteria to fail to grow and reproduce normally, thereby exerting a bacteriostatic action.

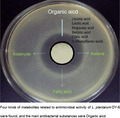

## 1 INTRODUCTION

Lactic acid bacteria have been widely used in food and feed because of their good antibacterial activity and safety. The antibacterial substances in previous studies were mainly organic acids (Reis, Paula, Casarotti, & Penna, 2012), fatty acids (Ogawa et al., 2005), short peptides (Hati, Patel, Sakure, & Mandal, 2017; Muhialdin, Zaiton, & Nazamid, 2018), and other categories. However, different lactic acid bacteria were found to possess specific combinations of antibacterial substances. The antibacterial substances of *Lactobacillus plantarum* LB1 were identified as lactic acid, benzene lactic acid, and formic acid (Rizzello, Cassone, Coda, & Gobbetti, 2011). It was reported that six organic acids, including formic acid, acetic acid, and propionic acid, produced by *Lactobacillus*, inhibited the growth of mold (Corsetti, Gobbetti, Rossi, & Damiani, 1998). Numerous studies have found that fatty acids also have antibacterial properties, such as antibacterial 3‐hydroxy fatty acid from *L. plantarum* MiLAB 14 (Schnürer, Sjögren, Kenne, Magnusson, & Broberg, 2003). The 2‐hydroxy‐4‐methyl‐n‐pentanoic acid in the fermentation broth of *L. plantarum* and *Lactobacillus wiesei* played a key role in inhibiting mildew (Ndagano, Lamoureux, Dortu, Vandermoten, & Thonart, 2011). And the bacteriocin paracin C produced by *L. paracasei* could effectively inhibit *Alicyclobacillus* (Pei, Yuan, & Yue, 2013). Though these substances were found to possess crucial antibacterial activity, due to the diverse metabolites of lactic acid bacteria, the specific antibacterial mechanism of each strain is deserved to discover.

The antimicrobial mechanisms of lactic acid bacteria were complicated. Some researchers have indicated that the organic acids produced by the fermentation process could reduce the intracellular pH by entering the cytoplasm, thus affecting the metabolism of pathogenic bacteria (Brul & Coote, 1999). In addition to lowering the intracellular pH, researchers also believed that organic acids could bind to some components, such as lipopolysaccharides on the cell membrane, thereby destroying the stability of the membrane and achieving bacteriostatic action (Gong et al., 2016). Moreover, it was found that phenyllactic acid could inhibit the protein expression of filamentous fungi (Ström, Schnürer, & Melin, 2005). The fatty acid was found to penetrate the pathogenic cells and bind to its plasma membrane, which then changed the permeability of the membrane to achieve bacteriostatic action (Bergsson, Arnfinnsson, Steingrímsson, & Thormar, 2001). Bacteriocin, isolated from lactic acid bacteria, acts as an antibacterial by destroying the integrity of the outer membrane of the pathogen (Khalaf et al., 2016).

Recently, technological breakthroughs in metabolomics have allowed for progress in the research of metabolic pathways of lactic acid bacteria, as well as the monitoring and optimization of the fermentation processes. GC‐MS was used to evaluate the changes of flavor substances in *Lactobacillus* plant during the fermentation of kimchi, and the quality of kimchi could be controlled effectively (Park et al., 2016). The ^1^H‐NMR technique was used to analyze metabolites in fermented soybean milk prepared by *Bifidobacterium* and *Streptococcus*, the factors influencing free radical scavenging activity of fermented soybean milk were expounded (Yang et al., 2009). *Lactobacillus plantarum* DY‐6 was found to have strong antibacterial activity; however, its antibacterial mechanism is not clear. Therefore, the antibacterial substances were evaluated and predicted by metabolomics, and the mechanism of antimicrobial substances was also explored in this study. We used GC‐MS and statistical methods to seek potential antibacterial substances and found that the antibacterial substances of *L. plantarum* DY‐6 were mainly organic acids and fatty acids, among which lactic acid and acetic acid showed excellent bacteriostatic activity. We also found that the antibacterial substances inhibited the growth of bacteria by destroying the stability of the cell membrane of pathogenic bacteria. The antibacterial substances could be quickly predicted by analyzing the metabolites produced by lactic acid bacteria and statistical analysis.

## 2 MATERIALS AND METHODS

### 2.1 Strains and media

*Lactobacillus plantarum* DY‐6 (CCTCC2017138), *L. casei* DY2 (CCTCC2017303), *L. rhamnose* DY4 (CCTCC2017279) and *Pediococcus acidilactici* DY5 (CCTCC2017280) were obtained from the China Center for Type Culture Collection. *L. rhamnose* DY3, *L. plantarum* DY1 and *L. plantarum* DY7 were preserved in our laboratory. All strains of lactic acid bacteria were cultured in deMan Rogosa Sharpe broth (Khalaf et al., 2016) at 37°C for 48 hr. The *Escherichia coli* ATCC25922, *Staphylococcus aureus* ATCC25923 and *Salmonella typhimurium* ATCC14028 were cultured in LB broth at 37°C for 24 hr.

### 2.2 Preparation of CFS

*Lactobacillus plantarum* DY‐6 was firstly incubated in MRS broth at 37°C for 24 hr. Bacterial culture broth was then centrifuged at 10,000 *g* for 15 min. CFS was filtered before use. Concentration of the supernatant was achieved by freeze‐drying.

### 2.3 The determination of antibacterial ability

Petri dishes, Oxford cups, and other materials were sterilized in an autoclave before use. Firstly, the bacterial suspension (10^8^ cfu/ml) was coated evenly on Luria‐Bertani agar medium prepared in a Petri dish. Then, oxford cups were placed on the surfaces of the cultures, and CFS was added by dripping. Finally, the Petri dishes with the Oxford cups were incubated at 37°C for 18 hr, and the diameter of the bacteria inhibiting loop was evaluated to indicate the antibacterial activity of the different samples (Wang, Zheng, Li, Wu, & Xiao, 2017).

### 2.4 Effects of temperature, pH, and enzyme on antibacterial activity

The antibacterial activities of CFS after the treatment of temperature, pH, and enzymes were determined by the Oxford cup method. CFS was treated for 30 min at temperatures of −20, 4, 30, 60, 80, 100, and 121°C, respectively. The change of antibacterial activity of CFS was tested. The pH value of CFS was adjusted to 2.0, 3.0, 4.0, 5.0, 6.0, 7.0, and 8.0 with 1 M HCl and 1 M NaOH and adjusted back to the initial pH of CFS after maintaining the pH for 1 hr. The antibacterial activity of CFS was then detected.

Separate CFS samples were treated with trypsin, pepsin, or proteinase K. The enzyme solution was adjusted to a concentration of 1 g/L and adjusted to the optimum pH for the enzyme: pepsin (2.0), trypsin (7.0), and proteinase K (7.5). The enzyme solution and the supernatant were mixed in equal volumes, and the antibacterial activity was measured. Before evaluating the antibacterial activity, the pH value of CFS was readjusted to the initial pH. The CFS without enzyme treatment was served as a control.

### 2.5 Determination of organic acids

Organic acids were detected using a Hitachi High‐Performance Liquid Chromatograph equipped with a UV absorption detector column. Ion chromatography was performed using an organic acid column. The mobile phase was 5 mM sulfuric acid solution at a flow rate of 0.6 ml/min and a column temperature of 50°C. The content of various organic acids was calculated according to the peak time and peak area (Zhao et al., 2018).

### 2.6 GC‐MS analysis of the metabolites

Cultures of *L. plantarum* DY‐6 were harvested during the platform period and centrifuged at 14,000 *g* for 15 min. The CFS was extracted with ethyl acetate. The extraction phase was dried by a nitrogen blower and derivatized at 85°C in 5 μl pyridine and 50 μl of N,O‐bis trimethylsilyl trifluoroacetamide for 20 min (Park et al., 2016).

One microliter of the derivatized sample was injected into a series 8000 GC on a TG‐5 column (30 m × 0.25 mm × 0.25 μm) with a split injection of 1:5. Helium was used as the carrier gas with a flow rate of 1 ml/min. The initial column temperature was 40°C, which was maintained for 2 min. The temperature was then increased from 40 to 280°C at a rate of 10°C/min. The source temperature was set to 280°C and the interface temperature was 300°C. Electron impact (EI) spectra were obtained at 70 eV.

### 2.7 Multivariate statistical analysis

All GC‐selected peaks were searched and identified using the NIST Mass Spectral Library (Tao & Zhang, 2010), and the peak area normalization method was used to calculate the relative percentage of each component. The collected data were processed using Simca14.1 software for principal component analysis (PCA) to observe the total metabolic differences (Becerra, Odermatt, & Nopens, 2013; Xu et al., 2016).

### 2.8 Effect of CFS on the growth curve of indicator bacterium

Two milliliters of *E. coli* suspension was added to 100 ml LB medium, and 2 ml sterile water was added as a control group at the initial stage of the culture. To the test group, 2 ml CFS was added, which was concentrated by 15 times at the initial stage of the culture. Other experimental groups had 2 ml of CFS added to the samples, which was concentrated 15 times after *E. coli* was cultured for 2, 4, 6, and 8 hr. A growth curve was produced for *E. coli* after treatment with CFS.

### 2.9 Effect of CFS on the content of soluble total sugar in indicator bacterial liquid

The content of total soluble sugar in the indicator bacterial solution was determined following the anthrone method (John, Barnett, & Miller, 2010). A suspension of *E. coli* was added in 1 g/L glucose solution, and CFS with a volume fraction of 2%, which was concentrated 10 times, was added to the experimental group. An equal amount of sterile water was added as a blank control. After treatment at 37°C for 0, 2, 4, 6, 8, 10, and 12 hr, the solution was centrifuged for 5 min at 5,000 *g* to obtain supernatant. The total soluble sugar of the supernatant was detected.

## 3 RESULTS

### 3.1 Comparison of the antibacterial activities of different lactic acid bacteria

The antibacterial abilities of seven lactic acid bacteria were determined (Table 1). According to the bacteriostasis of each strain on *E. coli*, *S. aureus* and *S. typhimurium*, among the seven bacteria indicated in Table 1, *L. plantarum* DY‐6 has the highest antibacterial activities; thus, it was selected for the next round of experiments. Besides, the results also indicated that all the three *L. plantarum* strains had excellent antibacterial activities (Table 1).

**TABLE 1:** Bacteriostatic effect of different lactic acid bacteria on pathogenic bacteria^a^

Strain number	Indicator organism		
	*Escherichia coli*	*Salmonella*	*Staphylococcus aureus*
*L. plantarum* DY1	12.89 ± 0.21	15.93 ± 0.22	15.70 ± 0.41
*L. casei* DY2	10.25 ± 0.23	10.72 ± 0.37	12.10 ± 0.29
*L. rhamnose* DY3	13.43 ± 0.39	11.57 ± 0.26	13.83 ± 0.35
*L. rhamnose* DY4	13.22 ± 0.14	12.20 ± 0.30	13.55 ± 0.38
*P. acidilactici* DY5	12.81 ± 0.22	11.60 ± 0.34	13.77 ± 0.22
*L. plantarum* DY6	15.32 ± 0.28	13.18 ± 0.19	16.08 ± 0.31
*L. plantarum* DY7	13.79 ± 0.33	12.40 ± 0.48	14.21 ± 0.17

^a^ Data are presented as the mean value ± standard deviation of three measurements.

### 3.2 Effects of temperature, pH, and enzyme on antibacterial activity of *L. plantarum* DY‐6

The effects of temperature, pH and enzymes addition on antibacterial activity of *L. plantarum* DY‐6 supernatant were determined using *E. coli* as an indicator strain. The data indicated that the temperature below 100°C had no significant effect on the antibacterial ability, while it was notably decreased when the temperature reached up to 121°C (Figure S1A). The supernatant only had antibacterial activity under acidic conditions, and it was gradually reduced from pH 2.0, (Figure S1B). In the present study, pepsin, trypsin, and proteinase K enzymes were used to treat the supernatant, and it was found that these enzymes had little effect on the antibacterial activity, indicating that the protein in the supernatant had little effect on antibacterial activity (Table 2).

**TABLE 2:** Effects of various proteinase on antibacterial activity after enzyme treatment^a^

Enzyme	Inhibition zone (mm)
Control^b^	14.10 ± 0.21
Trypsin	13.82 ± 0.26
Pepsin	13.71 ± 0.38
Proteinase K	13.90 ± 0.50

^a^ Data are presented as the mean value ± standard deviation of three measurements.

^b^ Fermentation supernatant without enzyme treatment.

### 3.3 Differential metabolite analysis in CFS

**FIGURE 1:**
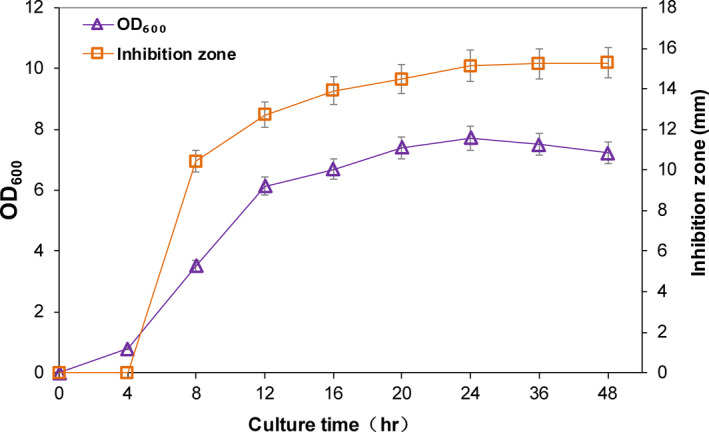
Growth curve of *Lactobacillus plantarum* DY‐6 and changes in antibacterial activity during fermentation

*Lactobacillus plantarum* DY‐6 entered the logarithmic growth phase from 4 hr, and its growth began to plateau after 24 hr. During this process, the antibacterial activity of CFS was enhanced with the time increase (Figure 1). At the initial stage (0–4 hr), CFS showed no obvious antibacterial activity, and then, it was significantly improved. The antibacterial activity tended to be stable after 24 hr (Figure 1), indicating that the antibacterial effect of the CFS was positively correlated with the growth of the bacteria.

**FIGURE 2:**
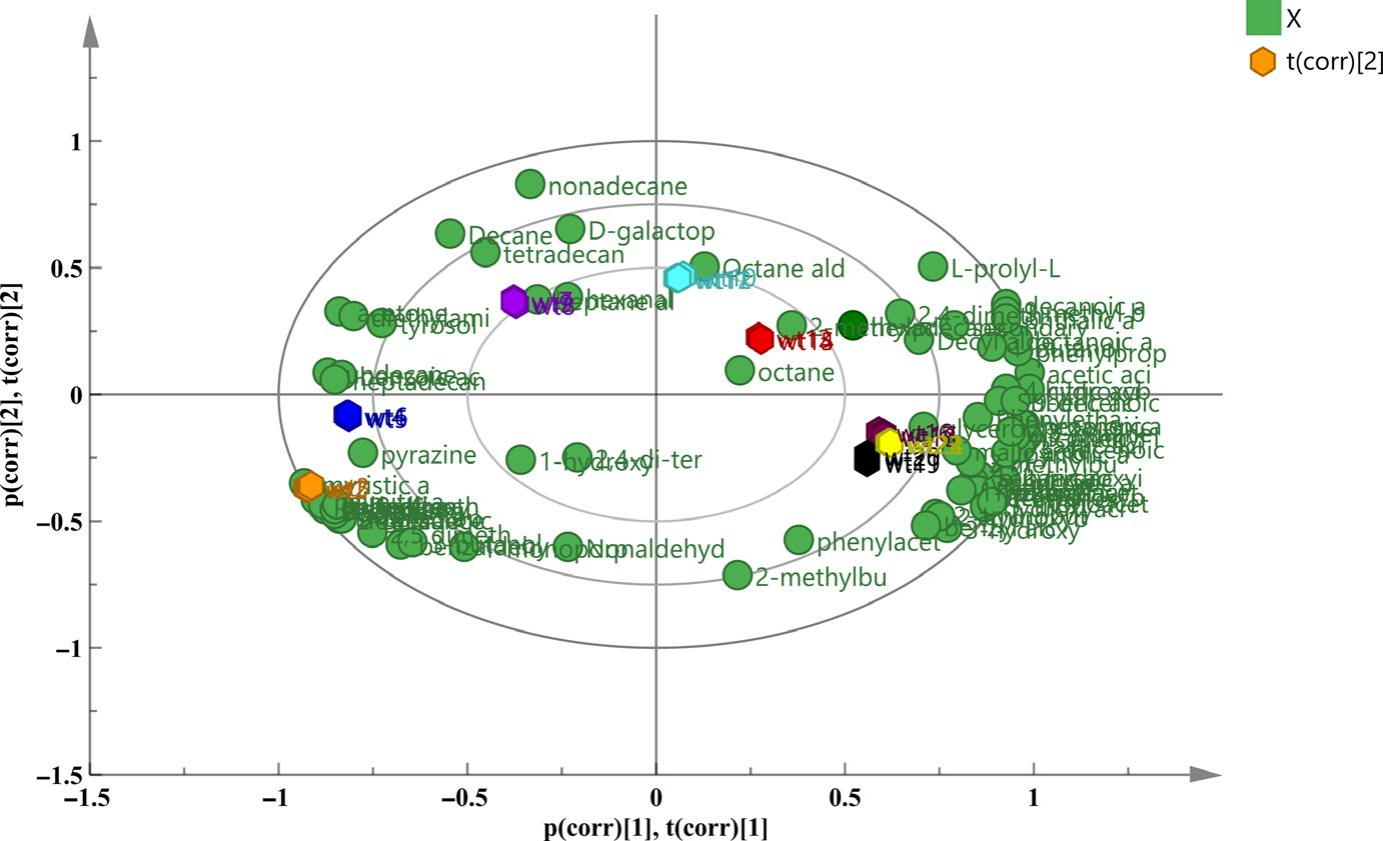
PCA scores and loadings biplot of the metabolites of CFS at different fermentation times. The symbols in the figure represent metabolites. The hexagonal patterns represent the fermentation time; from left to right, the times are 0, 4, 8, 12, 16, 36, 24, and 48 hr

**FIGURE 3:**
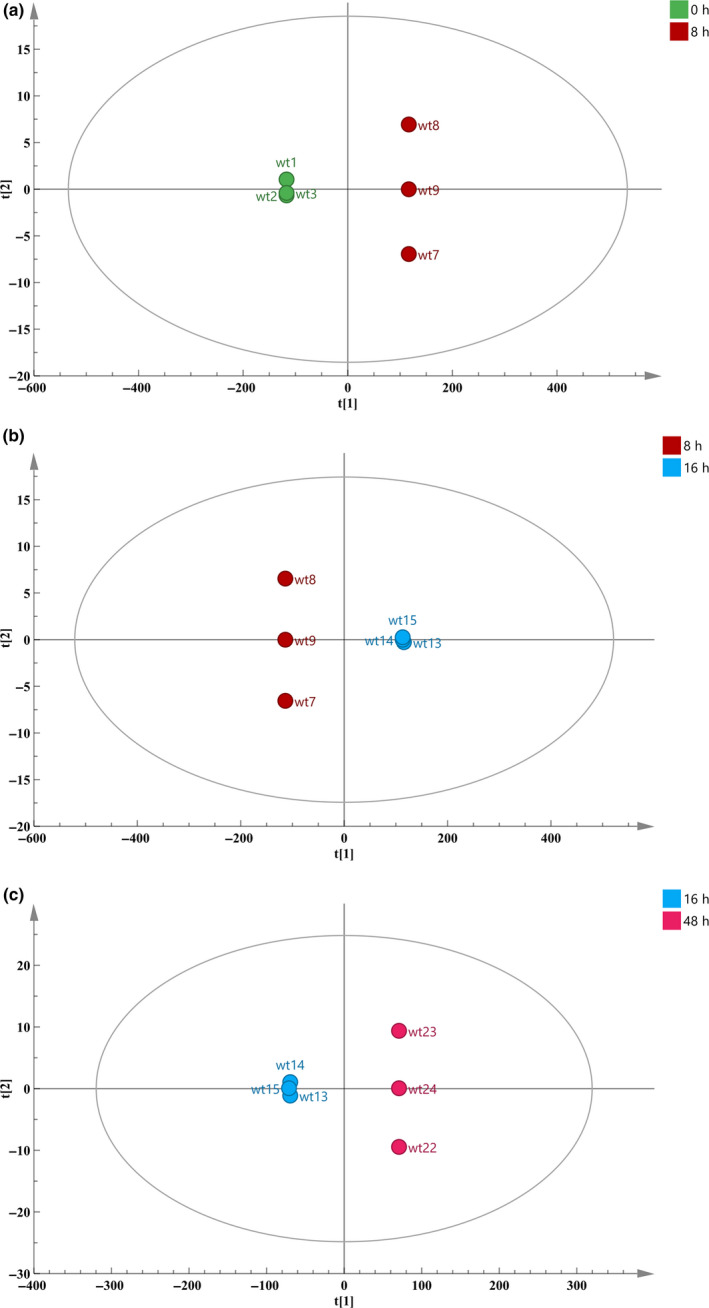
OPLS‐DA diagram of different fermentation time. a: 0 and 8 hr; b: 8 and 16 hr; c: 16 and 48 hr. The larger the distance between the two times, the greater the difference between the metabolites in their CFS

A PCA was used to analyze the sample data of different fermentation times. In this study, the entire fermentation process was divided into eight time periods according to their distance, and the farther apart, and the higher the degree of separation. Throughout the model, it was found that different time periods had a certain separation trend (Figure 2). Three groups of significantly different samples (0 and 8 hr; 8 and 16 hr; and 16 and 48 hr) were selected for analysis in combination with the antibacterial activity of different time periods (Figure 1) and the separation of each stage under this model (Figure 3a–c). Among the three selected groups, the two time periods in each group had a good separation trend and showed a large inhibition of bacteriostatic activity.

**FIGURE 4:**
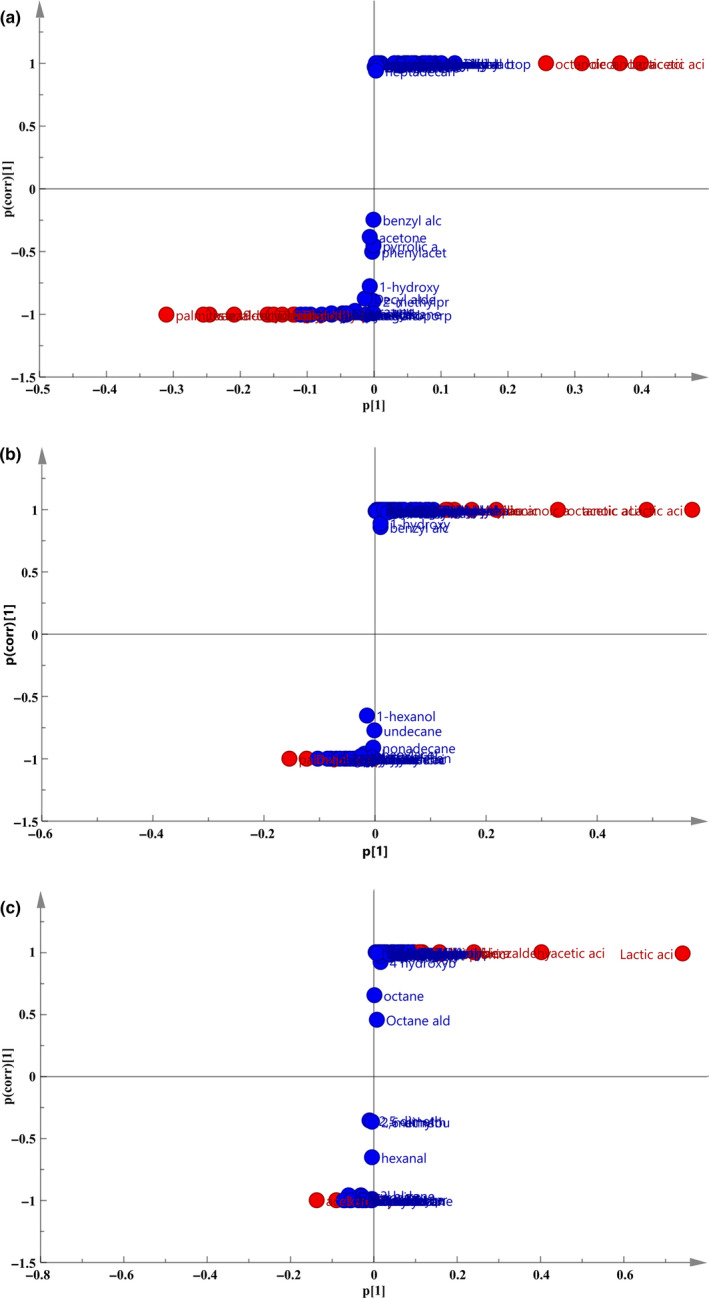
S‐plot diagram of different fermentation time. a: 0 and 8 hr; b: 8 and 16 hr; c: 16 and 48 hr. Each point in the diagram represents a metabolite. The farther the point in the figure is from the center, the more significant the change in metabolites

**FIGURE 5:**
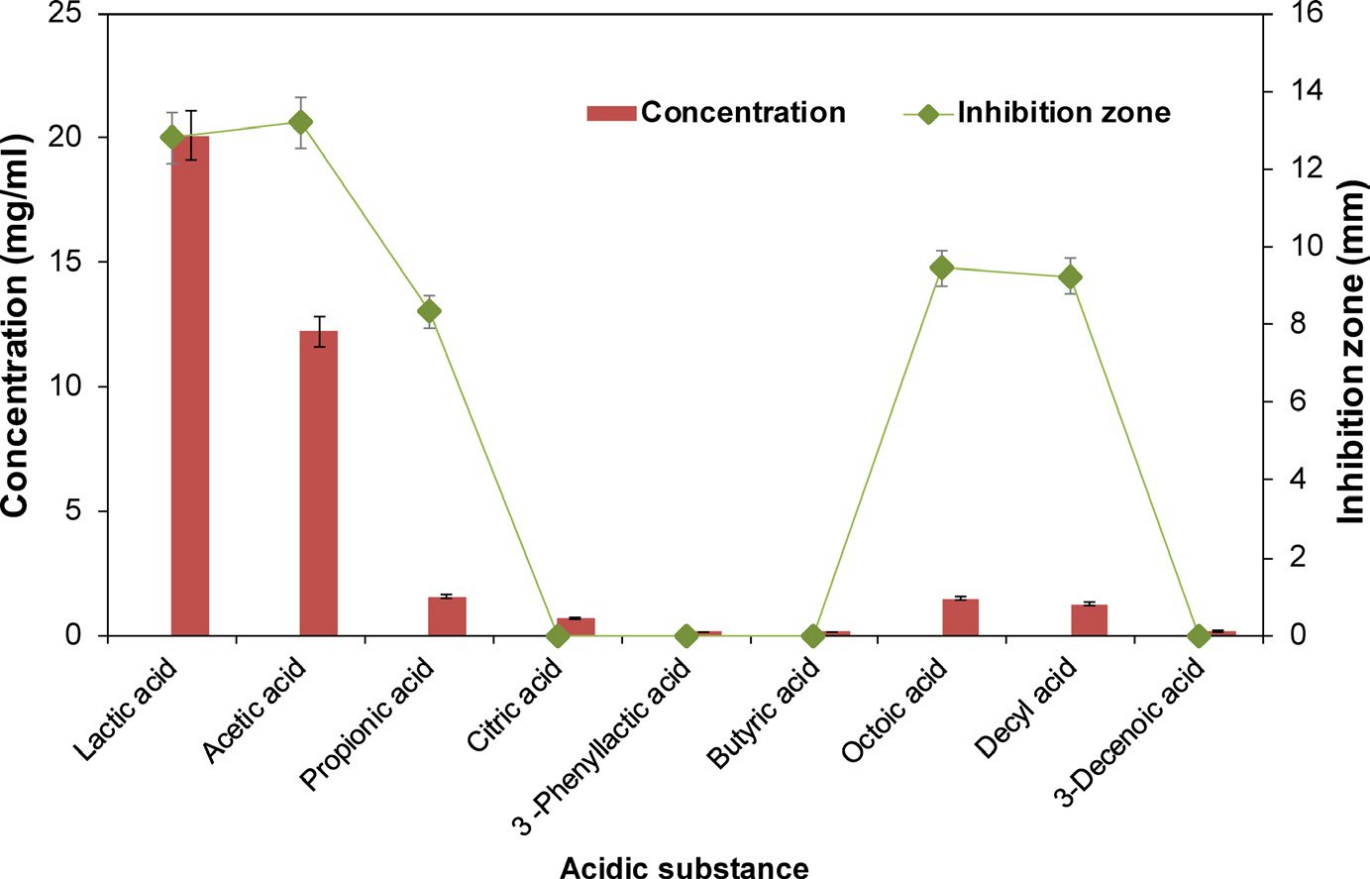
Antibacterial activity of acidic substances in CFS. 108 cfu/ml bacterial suspension and CFS were incubated at 37°C for 18 hr, and the diameter of the bacteria inhibiting loop was evaluated to indicate the antibacterial activity of the different samples (see Section 2)

The OPLS‐DA model was used to screen metabolites with significant changes between samples. Each dot in the figure represented a metabolite. The further the point in the figure from the center was, the more significant the change in this metabolites was. Differential variables were initially identified by selecting a variable with a VIP value >1 (marked in red in the Figure 4a–c). By analyzing the three groups of samples, thirteen types of metabolites which might have antibacterial activities were obtained, among which acid substances accounted for the vast majority (a total of nine types) (Figure 5). The other four substances were aroma substances from the fermentation process, but the content of these substances was low. Therefore, it was speculated that acidic substances played a major role on antibacterial activity in CFS.

In Figure 5, the contents of lactic acid, acetic acid, propionic acid, decanoic acid, and octanoic acid were relatively higher than that of other acidic substance, and they all showed antibacterial activity against *E. coli*, among which lactic acid and acetic acid showed superior bacteriostatic activity. Then, the above five antibacterial substances were compounded, and the antibacterial activity of the mixture was found to be similar with that of CFS, suggesting that these five substances were the main antibacterial substances in CFS.

### 3.4 Effect of CFS on the growth of indicator bacteria

**FIGURE 6:**
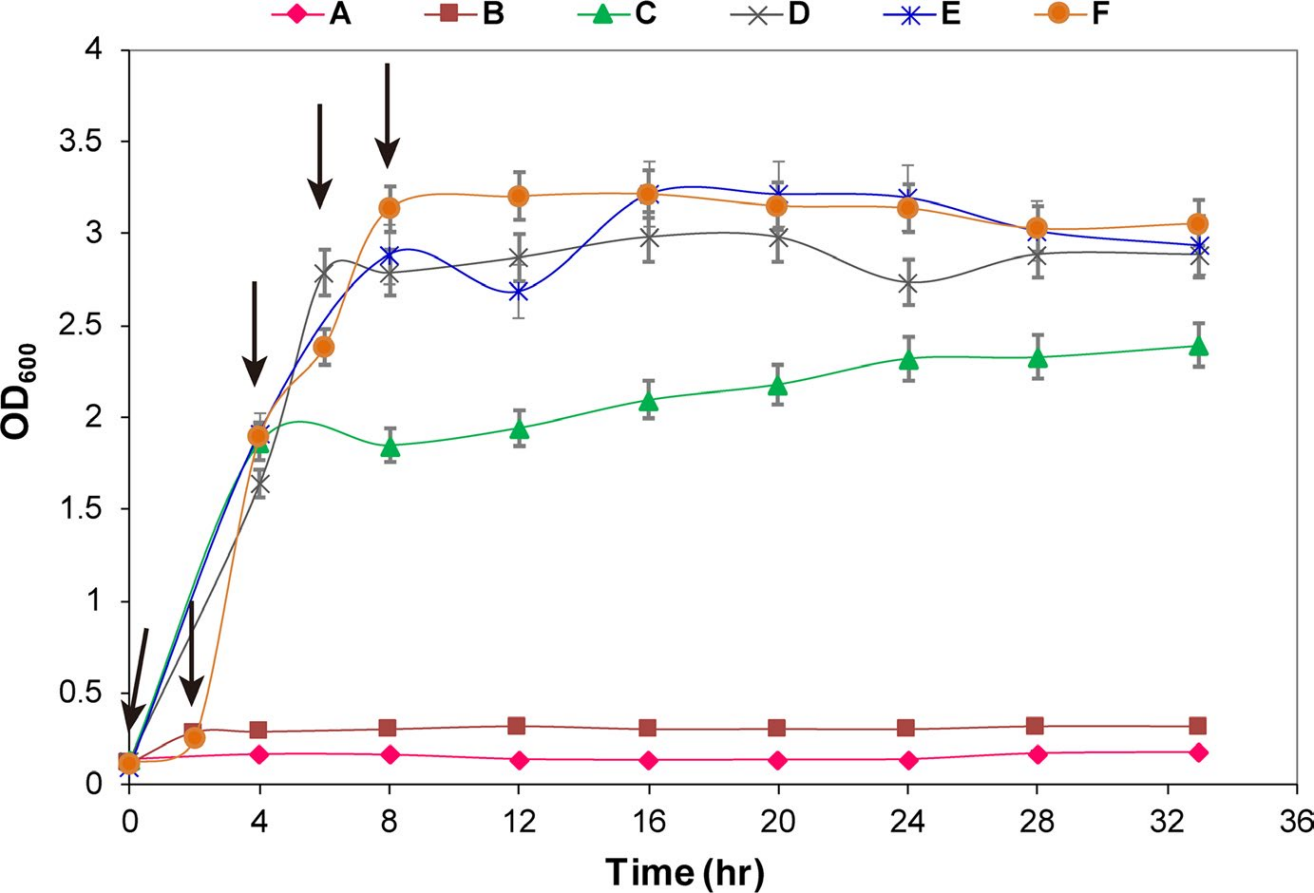
Effect of CFS on the growth curve of *E. coli*. CFS. A 2 ml CFS was concentrated 15 times and was added at 0, 2, 4, 6, or 8 hr; the black arrow represents the time when CFS was added (A: 0 hr, B: 2 hr, C: 4 hr, D: 6 hr, E: 8 hr, F: control)

The growth of *E. coli* was significantly affected, even did not grow, when CFS was added around 0–4 hr (Figure 6). However, the concentration of the cells differed little from that of the control group when CFS was added around 6–8 hr (Figure 6). The reason for the inhibition around 0–4 hr might be that the acidic growth environment derived from the addition of CFS, thus affecting the normal growth. And adding CFS had little effect when *E. coli* entered the late logarithmic phase after 6 hr.

### 3.5 Effect of CFS on total sugar content in indicator bacteria

Carbohydrates were the primary carbon source of microorganisms. When the membrane structure was destroyed, the cell contents will be leaked (Júnior et al., 2018). The structural integrity of the cell membrane can be judged by measuring the change in the sugar concentration (Yao, Li, Bi, & Jiang, 2015).

**FIGURE 7:**
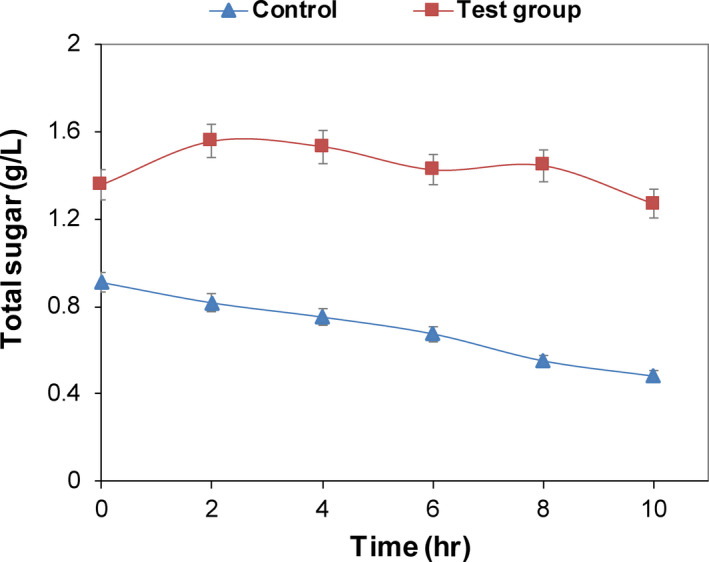
Effect of CFS on total soluble sugar in *E. coli* suspension. CFS with a volume fraction of 2% was concentrated for 10 times and then was added to the experimental group. After treatment at 37°C for 0, 2, 4, 6, 8, 10, and 12 hr, the solution was centrifuged for 5 min at 5,000 *g* to obtain their supernatants. The total sugar content of *E. coli* was determined at the indicated time

The results showed that the total sugar content in the *E. coli* solution increased first after the addition of CFS, then kept almost steady, indicating that the cell membrane structure was destroyed, and the intracellular carbohydrates were extravasated. While the sugar content of the control group continually decreased because of the carbohydrates utilization, indicating that CFS could interfere with the normal growth and metabolism of the cells (Figure 7).

## 4 DISCUSSION

In this study, we compared the antibacterial activity of lactic acid bacteria from different sample sources and found that *L. plantarum* DY‐6 had excellent antibacterial activity. *Lactobacillus plantarum* DY‐6 had a broad spectrum of inhibition, which could effectively inhibit *E. coli*, *S. aureus*, and *Salmonella*. The antibacterial properties of the fermentation supernatant were also investigated. The antibacterial substance had good thermal stability and antibacterial activity under acidic conditions. In addition, after the treatment with proteinase K, pepsin, and trypsin, the antibacterial activity did not change significantly, indicating that the protein substance played a limited role of antibacterial activity in CFS.

It was found that the antibacterial activity was closely related to the growth of the *L. plantarum* DY‐6. Thirteen different metabolites were found in CFS using GC‐MS combined with multivariate statistics. Among them, lactic acid, acetic acid, propionic acid, caprylic acid, and decyl acid exhibited good antibacterial activity. The antibacterial activity of CFS mainly came from these five substances. Others metabolites, such as phenyllactic acid and citric acid, have previously been shown to have antibacterial activity (Ning et al., 2017; Su et al., 2010), but their content in CFS was too low to achieve antimicrobial effect. Next, the effect of antibacterial substances on the growth of *E. coli* was explored. Antibacterial substances have an obvious inhibitory effect on the growth of *E. coli* and destroyed the stability of the membrane, causing the leakage of its cell contents, thus affecting the normal growth of bacteria.

The bacteriostatic action of lactic acid bacteria involves various metabolites. For example, acetic acid, formic acid, propionic acid, and butyric acid produced from lactic acid bacteria had a synergistic effect on molds (Su et al., 2010), and phenyllactic acid derived from the culture of *L. plantarum* IMAU10014 was found to have bacteriostasis effects (Wang et al., 2012). Other antibacterial substances isolated from *L. plantarum*, such as 3‐hydroxydecanoic acid and decanoic acid, had good antibacterial activity on fungi (Guo et al., 2012). Several antibacterial bacteriocins have also been previously isolated and extracted (Camargo, Todorov, Chihib, Drider, & Nero, 2018; Kaškonienė et al., 2017; Langa, Arqués, Medina, & Landete, 2017). In addition, hydrogen peroxide and other substances produced during the fermentation process were also shown to have antibacterial activity (Atanasova, Moncheva, & Ivanova, 2014; Castillo et al., 2002).

The antibacterial mechanisms of organic acids in metabolites were extensively studied. The common mechanism was to inhibit the stability of the membrane or reduce the intracellular pH, thereby affecting the metabolic activities of pathogenic bacteria, achieving inhibition. The bacteriostatic mechanism of fatty acids produced during fermentation was similar to that of organic acids. The bacteriocin activity could increase the permeability of cell membranes, causing leakage of various ions in the cells and consumption of energy substances, and finally resulting in the death of pathogenic bacteria. Recently, Piewngam et al. (2018) found that lipopeptides produced by *Bacillus* could affect the quorum sensing system of the antigen body and then interfere with their colonization to achieve bacteriostatic action, which also provided a new perspective to study the bacteriostatic action of lactic acid bacteria.

To summarize, the bacteriostatic action of lactic acid bacteria was closely related to its metabolites, and the metabolites of different lactic acid bacteria are different. We could infer the possible bacteriostatic substances by analyzing the total metabolites of lactic acid bacteria. We were able to accurately identify the antibacterial products, which provided a good foundation to study the antibacterial effect of lactic acid bacteria.

## 5 CONCLUSIONS

A strain of *L. plantarum* DY‐6 with excellent antibacterial activity was studied. The antimicrobial activity of *L. plantarum* DY‐6 is closely related to its metabolites. The main antibacterial substances were found to be lactic acid, acetic acid, propionic acid, caprylic acid, and decyl acid. The active antibacterial substances produced by the lactic acid bacteria destroyed the cell membrane structure of the bacteria, causing bacteria to fail to grow and reproduce normally, thereby exerting bacteriostatic action. This study provides an effective method for rapid screening of antimicrobial substances.

## CONFLICT OF INTEREST

The authors declare that the research was conducted in the absence of any commercial or financial relationships that could be construed as a potential conflict of interest.

## Supporting information

Figure S1
