# Supplementary material for: Identification of antibacterial substances of Lactobacillus plantarum DY‐6 for bacteriostatic action
Source: Food Sci Nutr. 2020 May 12;8(6):2854–63. doi: 10.1002/fsn3.1585 (PMC7300085; doi:10.1002/fsn3.1585)
Supplement: Supplementary file 1 — Figure S1 [file FSN3-8-2854-s001.doc]

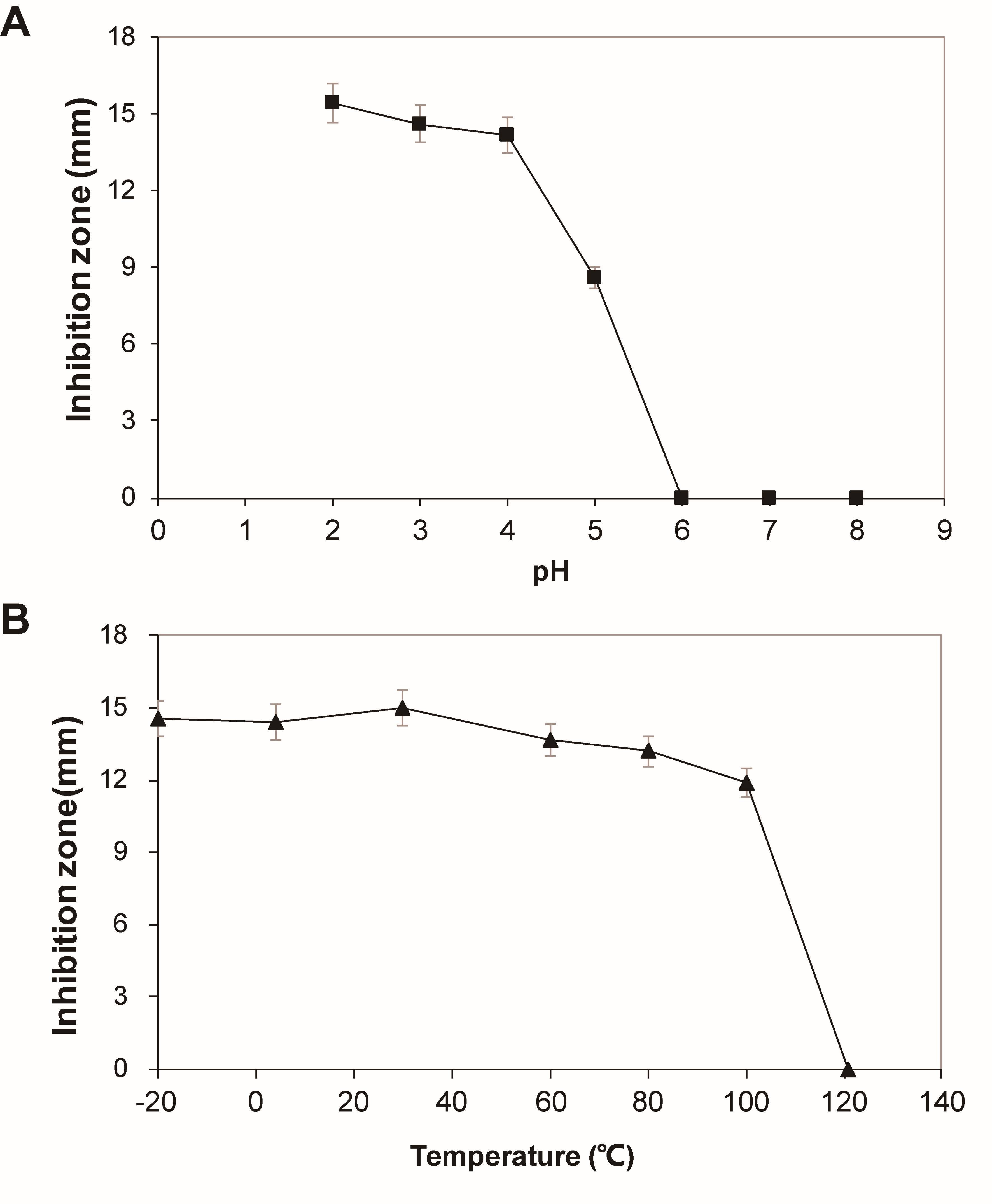


**Supplementary Figure 1**. Effects of various temperatures (A) and pH values (B) on the antibacterial activity.
